# Distal Arch Aneurysm Discovered With Dysphagia

**DOI:** 10.7759/cureus.43406

**Published:** 2023-08-13

**Authors:** Hideki Sasaki, Yukihide Numata, Shinji Kamiya, Yoshiaki Sone, Syunta Hayakawa

**Affiliations:** 1 Cardiovascular Surgery, Nagoya City University East Medical Center, Nagoya, JPN

**Keywords:** contrast-enhanced computed tomography, vascular compression, bronchial artery, hoarseness, recurrent laryngeal nerve paralysis, dysphagia, distal arch aneurysm

## Abstract

A 64-year-old man sought medical attention from a family physician, expressing concerns about dysphagia. Recognizing the complexity of the symptoms, the family physician promptly engaged the expertise of an attending physician at a regional hospital to ensure accurate diagnosis and management. Plain computed tomography (CT) revealed a space-occupied lesion located posterior to the trachea. Although mediastinal tumor was suspected at first, contrast-enhanced CT revealed a distal arch aneurysm that compressed the esophagus. The patient underwent total arch replacement, and the postoperative course was uneventful.

## Introduction

Although an aortic arch aneurysm is a life-threatening disease [1], patients usually do not present symptoms unless its diameter is rather large. Moreover, it is difficult for a family physician to detect it during a regular checkup. The symptom of dysphagia is non-specific and does not neessarily indicate an aortic disease [2]. Here, we present a case in which a mediastinal tumor was suspected at the regional hospital, but it turned out to be a distal arch aneurysm.

## Case presentation

**Figure 1:**
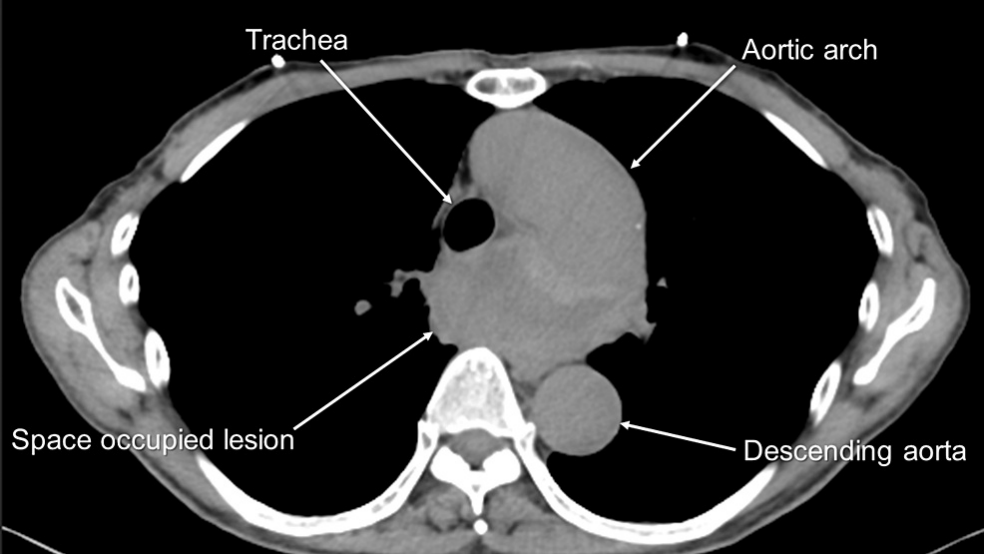
Plain computed tomography (CT) scan demonstrating a space occupying a lesion located posterior to the trachea

A 64-year-old man with a complaint of dysphagia visited a family physician. The family physician consulted an attending physician at a regional hospital. The attending physician checked the chest X-ray, which confirmed no lesions. Then, a plain computed tomography (CT) was ordered. Plain CT revealed a space-occupied lesion located posterior to the trachea (Figure 1).

**Figure 2:**
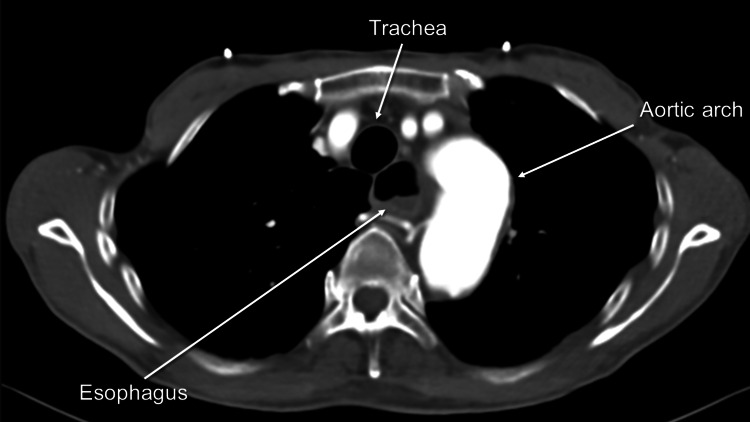
Contrast-enhanced computed tomography (CT) revealing dilated esophagus

**Figure 3:**
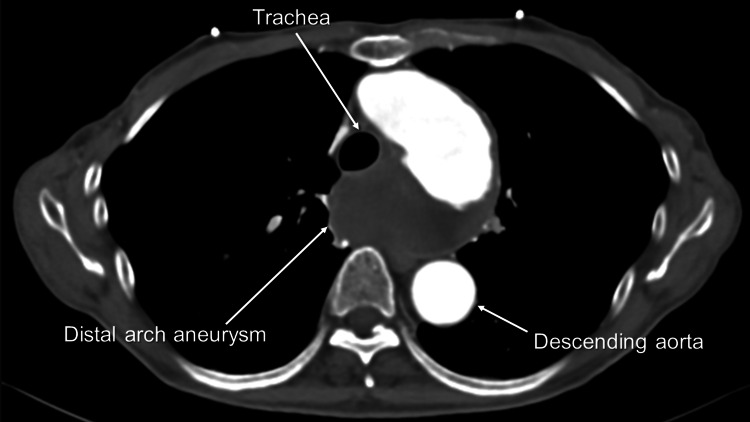
Preoperative contrast-enhanced computed tomography (CT) revealing a distal arch aneurysm

The attending physician suspected a mediastinal tumor and consulted the respiratory department. Contrast-enhanced CT revealed a distal arch aneurysm that compressed the esophagus whose lumen was dilated proximal to the aneurysm (Figures 2, 3).

**Figure 4:**
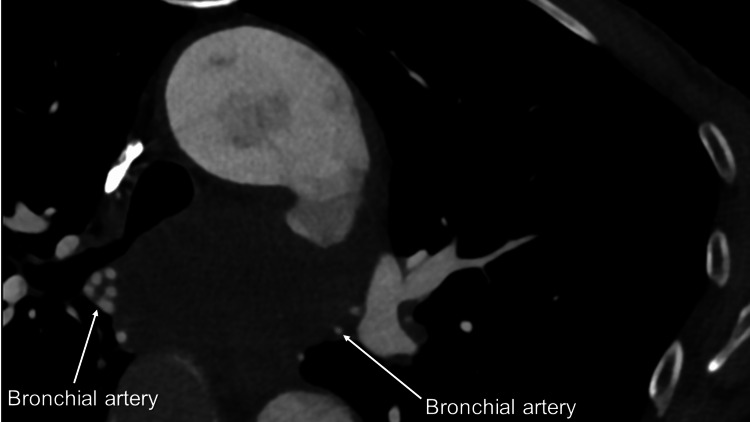
Preoperative contrast-enhanced computed tomography (CT) showing bronchial arteries around a distal arch aneurysm

**Figure 5:**
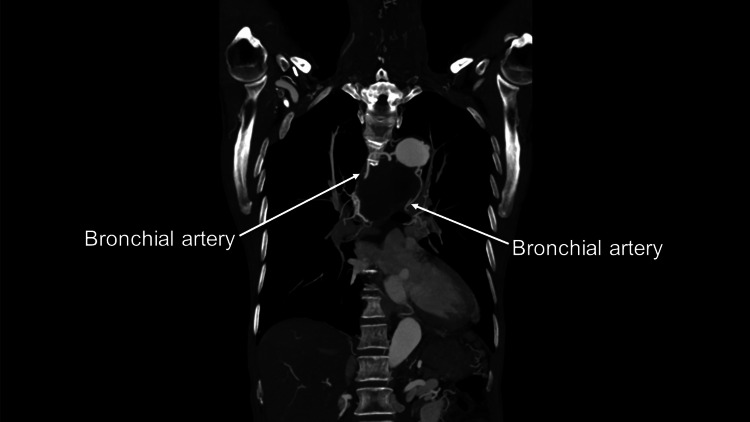
Preoperative contrast-enhanced computed tomography (CT) Better visualized bronchial arteries in the coronal view

In addition, relatively large bronchial arteries were found (Figures 4, 5).

The patient was referred to us for further treatment. The patient’s comorbidity was diabetes mellitus requiring an oral hypoglycemic agent, and hemoglobin A1C was 6.7%. The patient has been a smoker for 44 years. As the patient presented with hoarseness, he was consulted in the otorhinolaryngology department, and left recurrent laryngeal nerve paralysis (LRLNP) was found. After a careful assessment, we decided to perform an aortic arch surgery. Under general anesthesia, a median sternotomy was performed. Cardiopulmonary bypass (CPB) was established with ascending aortic perfusion and superior and inferior vena cava drainage. The patient was cooled down, and the circulatory arrest was induced at a rectal temperature of 22°C. To maintain cerebral perfusion, antegrade selective cerebral perfusion was initiated using balloon-tipped cannulas. After opening the aorta and aortic arch, we confirmed the distal arch aneurysm filled with thrombi.

**Figure 6:**
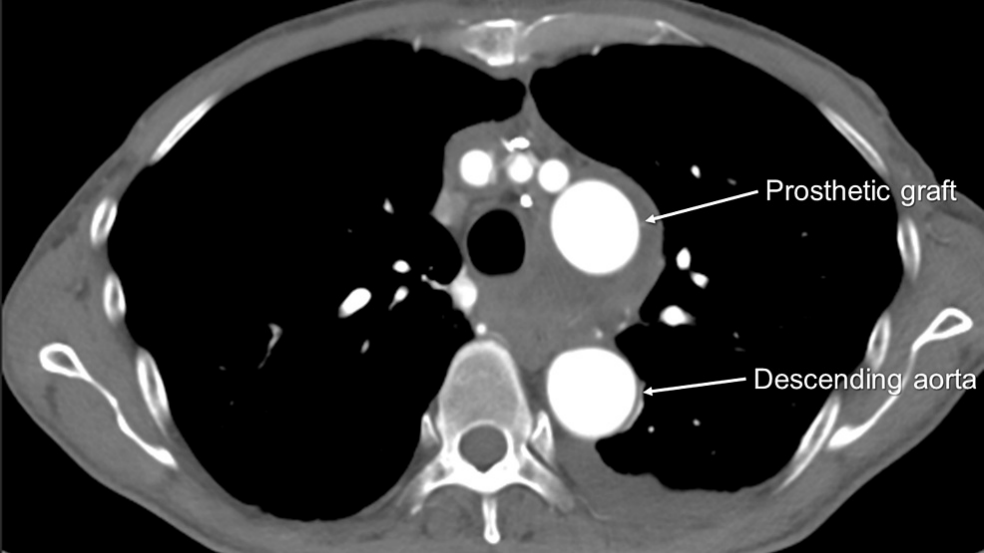
Postoperative contrast-enhanced computed tomography (CT) revealing a disappeared aneurysm

Furthermore, in creating the distal anastomosis site, we identified several bronchial arteries that originated from the distal arch. They were meticulously ligated or clipped to mobilized the distal aorta. The distal anastomosis site was created, and a 28 mm four-branched graft (J Graft, Japan Lifeline, Tokyo, Japan) was anastomosed. Subsequently, proximal aortic anastomosis and arch vessel anastomoses were completed. The patient was weaned from CPB without difficulty and transferred to the intensive care unit (ICU). Circulatory arrest, aortic cross-clamping, and CPB time were 79, 140, and 201 minutes, respectively. Twelve hours after ICU admission, the patient was weaned from the ventilator. Before removing the tracheal tube, we consulted the otorhinolaryngology department again. The otorhinolaryngologist performed laryngoscopy, which revealed that the left side of the vocal cord did not move. However, no edema was found on the epiglottis. Oral intake was started. The patient was transferred to the general ward for rehabilitation. The postoperative CT revealed a disappeared aneurysm (Figure 6), and the patient was discharged home on postoperative day 19. Currently, six months after the operation, hoarseness has improved.

## Discussion

Aortic arch aneurysm represents a rare yet formidable medical condition, fraught with life-threatening implications. In its early stages, the absence of symptoms prevails, unless the aneurysm attains a considerable diameter [2]. Hence, the criticality of discerning this condition accurately and administering appropriate therapeutic measures cannot be overstated. Within the context of this particular case, four salient points related to this condition warrant an in-depth discussion.

The first point recognizes that dysphagia emerges as a primary manifestation, despite its lack of specificity. It is uncommon as a manifestation of an aortic aneurysm [2]. The differential diagnosis for dysphagia encompasses an extensive array of plausible etiologies, including cerebrovascular disorders, such as brain infarction, brain hemorrhage, and subarachnoid hemorrhage; intricate neurological ailments, such as extrapyramidal disease, spinocerebellar degeneration, multiple sclerosis, amyotrophic lateral sclerosis; and peripheral nerve diseases, such as myodystrophy, multiple myositis, myasthenia gravis, and esophageal achalasia. Although a conscientious physician may contemplate some of these differential considerations upon evaluating a patient with dysphagia, prevailing clinical practice mandates the judicious inclusion of a plain CT scan to augment diagnostic precision. In light of the myriad potential differentials, the discerning physician must prioritize the expeditious exclusion of any emergent conditions. Thus, the prudent course of action involves ordering a plain CT scan in the incipient stages, safeguarding against perilous oversight and facilitating timely intervention.

The second point to consider is the importance of imaging studies, especially contrast-enhanced CT. Such imaging studies, like plain CT scans, are commonly ordered in outpatient clinics due to their ease and lack of specific preparation requirements. In this particular case, the attending physician at a regional hospital ordered a contrast-enhanced CT, which revealed a space-occupied lesion located behind the esophagus. Initially, the physician suspected a mediastinal tumor; however, the contrast-enhanced CT identified a distal aortic arch aneurysm instead. It highlights the significance of careful examination by physicians when interpreting presented imaging studies, enabling them to suspect the correct disease and proceed with the next steps for an accurate diagnosis.

The third point emphasizes the importance of recognizing bronchial arteries. Surgeons must accurately identify the location of the aneurysm, the position of arch vessels, the distance between the aneurysm and arch vessels, the presence of bronchial arteries, and the anatomical proximity of the esophagus. It is crucial for surgeons to thoroughly examine the aortic arch and surrounding organs, paying careful attention to the presence of any vessels in between. Preoperative evaluation of the aortic arch and its surrounding organs is essential to ensure a successful surgical procedure. During the operation, surgeons are advised to compare the actual number and position of bronchial arteries with those identified in preoperative contrast-enhanced CT scans. If any additional, previously unidentified bronchial arteries are found around the distal anastomosis site in the preoperative CT scans, the surgeons should ligate or clip them to ensure a safe and successful surgical procedure. Despite being relatively large in diameter in the current case, small bronchial arteries can be easily overlooked, leading to intractable bleeding. Once the heart resumes its rhythm, it becomes even more challenging to locate them. Utilizing a 3D CT angiogram can be helpful in recognizing the anatomical course of bronchial arteries, as they often have a tortuous path. Surgeons must be precise in determining the number of bronchial arteries through preoperative enhanced CT scans, as what may appear to be many could actually be two or three. There are typically two left and one right bronchial arteries [3]. Keeping this in mind during their assessment is crucial.

The fourth point involves the careful assessment of LRLNP and the prevention of aspiration pneumonia. The hoarseness is described as the initial symptom of aortic aneurysm [4]. One of the most crucial considerations is the evaluation of LRLNP and taking measures to avoid aspiration pneumonia. In the current case, the patient presented with hoarseness due to a large arch aneurysm. Otorhinolaryngology department was consulted before and after the operation. LRLNP was diagnosed on both occassions. No edematous epiglottis was observed. We proceeded cautiously with oral intake, and no aspiration pneumonia was detected. The hoarseness symptom has been showing improvement. Given that aspiration pneumonia is a serious complication [5], a meticulous assessment of the vocal cords is imperative after the operation.

## Conclusions

When encountering non-specific symptoms, such as dysphagia and hoarseness, a physician should consider various possible diseases and plan effective examinations to approach the diagnosis. An accurate and careful assessment of the presented imaging studies, such as not overlooking the bronchial artery, is important in planning the treatment. Even after the operation, the physician should remain vigilant and pay attention to possible pitfalls to avoid serious complications, such as aspiration pneumonia.
